# Biophysical characterisation of the novel zinc binding property in Suppressor of Fused

**DOI:** 10.1038/s41598-017-11203-2

**Published:** 2017-09-11

**Authors:** Amira Jabrani, Staëlle Makamte, Emilie Moreau, Yasmine Gharbi, Anne Plessis, Lucia Bruzzone, Matthieu Sanial, Valérie Biou

**Affiliations:** 1 Laboratoire de Biologie Physico-Chimique des Protéines Membranaires, UMR 7099 CNRS, Université Paris Diderot, Sorbonne Paris Cité, PSL Research University, Institut de Biologie Physico-Chimique, 13 rue Pierre et Marie Curie, 75005 Paris, France; 2 Institut Jacques Monod UMR 7592, CNRS, Université Paris Diderot, Sorbonne Paris Cité, F-75205 Paris, France

## Abstract

Suppressor of Fused (SUFU) is a highly conserved protein that acts as a negative regulator of the Hedgehog (HH) signalling pathway, a major determinant of cell differentiation and proliferation. Therefore, SUFU deletion in mammals has devastating effects on embryo development. SUFU is part of a multi-protein cytoplasmic signal-transducing complex. Its partners include the Gli family of transcription factors that function either as repressors, or as transcription activators according to the HH activation state. The crystal structure of SUFU revealed a two-domain arrangement, which undergoes a closing movement upon binding a peptide from Gli1. There remains however, much to be discovered about SUFU’s behaviour. To this end, we expressed recombinant, full-length SUFU from Drosophila, Zebrafish and Human. Guided by a sequence analysis that revealed a conserved potential metal binding site, we discovered that SUFU binds zinc. This binding was found to occur with a nanomolar affinity to SUFU from all three species. Mutation of one histidine from the conserved motif induces a moderate decrease in affinity for zinc, while circular dichroism indicates that the mutant remains structured. Our results reveal new metal binding affinity characteristics about SUFU that could be of importance for its regulatory function in HH.

## Introduction

The evolutionarily conserved Hedgehog (HH) pathway is crucial during multiple steps of animal embryo development as it controls a number of cell functions including division, survival, migration and differentiation. For example, HH controls the patterning of the wings and the appendages in flies, and the development of the limbs, nervous tube, brain, muscles and vessels in humans. In humans, dysfunction of HH is implicated in numerous pathologies such as developmental defects, cardiovascular diseases and cancers^1^.

To understand the mechanism underlying the function of HH, the Drosophila model has been essential. The HH protein binds to its co-receptors at the plasma membrane. One of them, the transmembrane receptor Patched (PTC), catalytically inhibits the “G-Protein Coupled Receptor -like” Smoothened (SMO) protein in the absence of HH^2, 3^. Once activated, SMO acts on an intracellular multi-protein complex, the Hedgehog Transducing Complex (HTC), whose composition varies according to the HH activation state^4–6^, but which contains and controls the zinc-finger transcription factor Cubitus interruptus (CI), which belongs to the Gli family. When the HH ligand is not bound to PTC, the HTC associates with microtubules^7, 8^. This triggers CI phosphorylation, ubiquitination and targeting to the proteasome^9–11^. As a result, CI is partially proteolysed into a shorter form that acts as a repressor towards HH target genes. On the other hand, when HH binds to PTC, its inhibitory effect on SMO is suppressed and SMO accumulates as a hyperphosphorylated form at the cell surface. Consequently, the HTC bound to the SMO C-terminal tail leaves the microtubules and is also recruited at the plasma membrane^12, 13^. CI cleavage is thus prevented, and full-length CI triggers the transcription of target genes in the nucleus^9, 14^.

In mammals, a key component of HH signalling is the pioneer protein Suppressor of Fused (SUFU). This protein was initially discovered in flies for its ability to suppress the reduction in HH signalling resulting from mutations in a protein kinase called Fused (FU), pointing to SUFU’s inhibitory function^15^. It was later shown to directly interact with FU and CI, and to be associated with the HTC in the cytoplasm^5, 6, 16^. It acts as an ultimate gatekeeper that limits the activity of CI in the absence of HH both via limiting the nuclear entry of uncleaved CI, and probably as a cofactor that modulates CI transcriptional activity^17–19^. In all organisms, SUFU is seen both in the cytoplasm and in the nucleus, is associated with CI/Gli, and seems to enhance Gli affinity to DNA^18, 20^.

There is a high-level of sequence conservation of SUFU: amino-acid sequence identity is 37.6% between *Drosophila melanogaster* and human, 36.3% between *Drosophila melanogaster* and Zebrafish (*Danio rerio*), and 81.2% between human and Zebrafish. Despite this high sequence conservation, SUFU removal in mammals and Drosophila has very contrasting effects: in flies, loss-of-function SUFU mutants do not exhibit a notable developmental or reproduction phenotype^15^, whereas SUFU deficient mice die before birth due to severe defects linked to HH upregulation^21, 22^. In zebrafish, the *dre* loss-of-function mutant affects SUFU and results in a point mutation replacing Thr 111 by a lysine. It triggers HH overactivation resulting in eye, ear and fin defects in the embryo and misformed gill reminiscent of lung defects in mice^23^. Regardless of those phenotypic differences, SUFU seems to have a conserved function across species. Drosophila SUFU can restore proper HH signalling in SUFU deficient mammalian cells^24^, and murine SUFU can interact with FUSED and CI from Drosophila, and can be regulated by HH in flies^25, 26^.

In Drosophila melanogaster, SUFU is a 468 amino acid, 53 kDa, protein composed of two highly conserved regions at the N- and C-termini (42% and 29% identity between human and fruit fly sequences, respectively), interposed by a less conserved region of about 80 amino acids. The recently published crystal structures of the human and Drosophila SUFU show that the protein has two alpha/beta domains arranged in an elongated shape^27, 28^.

Zinc is the second most abundant transition element in the human body and its electronic properties, with a saturated d electron shell, make it a very flexible binder with no particular preferential geometry, as well as a stable divalent ion that is not easily oxidised or reduced^29^. The role of Zinc is far more complex than its definition as an essential nutrient. It is a cofactor for many enzymes (for example carbonic anhydrase that catalyses zinc-dependent hydration of CO_2_ reviewed in ref. 30) and it is necessary for the structuration of transcription factors or other proteins such as Sonic hedgehog^31^ itself as a crystal structure revealed the presence of Zinc in its N terminal domain. Zinc homeostasis is regulated by transporters^32^ and is essential for signal transduction in the brain and in immunity (reviewed in refs 33 and 34). Still now, many things are unknown about zinc, in particular because it is spectroscopically silent in the UV-visible light range and therefore difficult to trace.

We present here the zinc binding properties of SUFU. Analysis of multiple sequence alignment revealed a conserved HWHY motif that is part of a pocket containing other conserved aminoacids in the N-terminal domain of SUFU. A search for protein structures with similar aminoacids suggested that this pocket might bind cations. In order to test if this was the case, we expressed and purified full-length SUFU from Drosophila, Zebrafish and Human, hereafter named dSUFU, zSUFU and hSUFU, respectively. We chose to express SUFU from these organisms because they represent important phyla in evolution, and have different requirements for SUFU and FUSED. Our spectroscopic studies demonstrate that SUFU does bind zinc in a pH-dependent manner, and with a nanomolar affinity. Mutation of the two histidines by alanines resulted in destabilisation of the protein for the first histidine and in a decrease in the zinc affinity for the second one.

## Results

### Structural characterisation of SUFU in solution using circular dichroism

Synchrotron Radiation Circular Dichroism (SRCD) spectra were collected at the DISCO beamline at the SOLEIL synchrotron.

#### CD spectra and thermal unfolding of dSUFU

**Figure 1:**
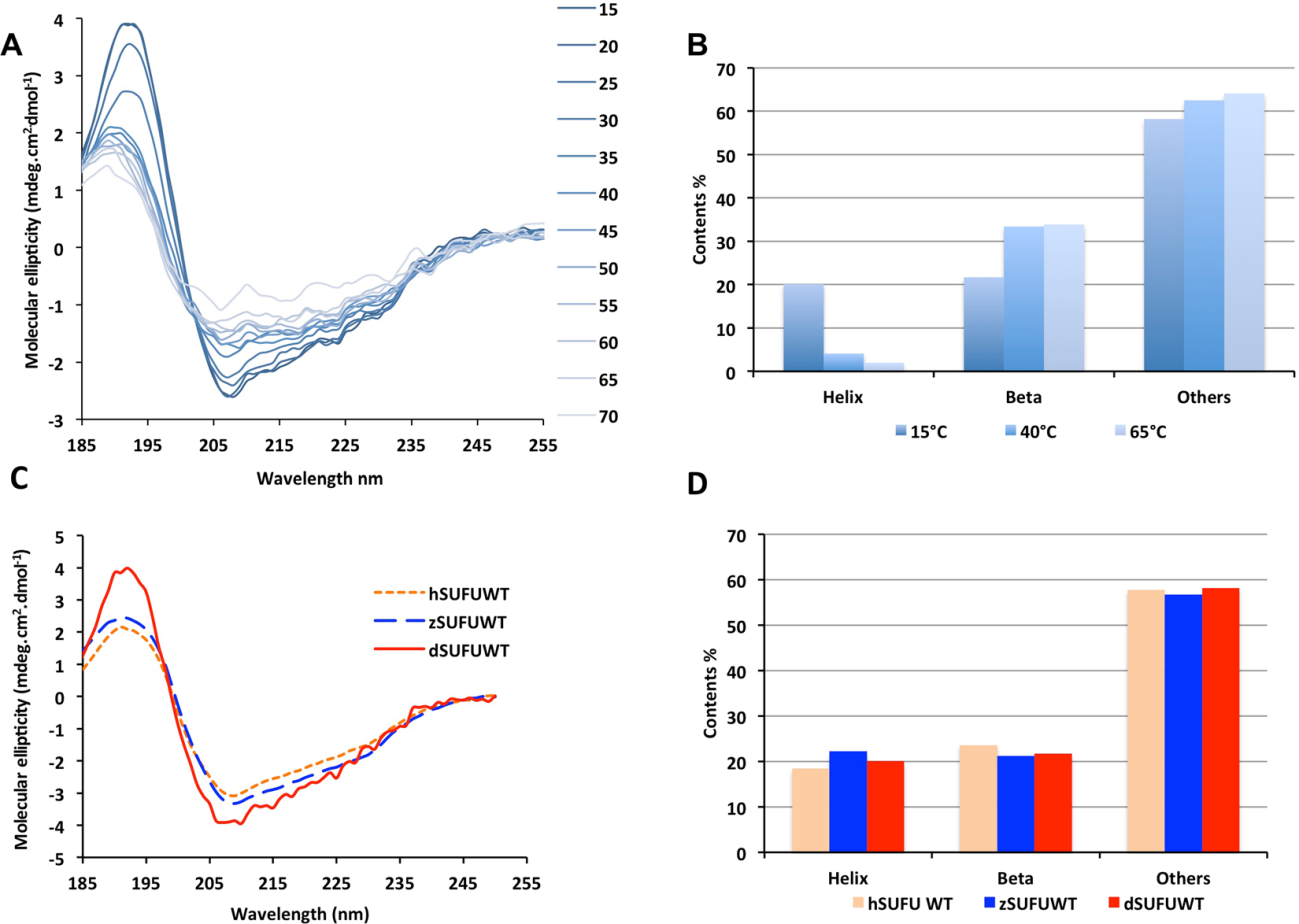
Circular dichroism studies of dSUFU show that it becomes richer in beta strand upon thermal unfolding and that SUFU from different species have similar secondary structure contents. (**A**) CD spectra of dSUFU in Hepes buffer at different temperatures; (**B**) Graph of secondary structure evaluation in Hepes buffer at three different temperatures; (**C**) CD spectra of hSUFU (orange dotted line), zSUFU (blue hashed line) and dSUFU (red solid line) at 15 °C; (**D**) Secondary structure estimation using Bestsel analysis of CD spectra of hSUFU, zSUFU and dSUFU (same color code as (**C**).

The SRCD spectrum of dSUFU at 15 degrees presents a maximum at 190 nm and a minimum at 208 nm (Fig. 1A). This is characteristic of a protein with α-helix, β strand and aperiodic secondary structures (i.e., “coil”). The BestSel method^35^ indicates that dSUFU CD spectrum is consistent with secondary-structure content of 20% helix and 22% ß-strand at 15 °C. This is in agreement with the crystal structure of the Drosophila protein 4KMA that contains 24% helix and 21% ß-strand (Table 1). SRCD spectra were measured as a function of increasing temperatures to characterise thermal unfolding of dSUFU (Fig. 1A). An isobestic point is present at 200 nm, indicating that a transition occurs between two states. The minima at 208 and 222 nm become shallower as the temperature rises suggesting a loss of α–helix in the structure of the protein. As analysed by the BestSel program, the thermal unfolding of SUFU indeed results in an increase in beta structure and a decrease in alpha-helix content, whereas turns and “other conformations”, i.e., aperiodic, remain almost constant (Fig. 1B). A gain in beta structure is rather frequent in many proteins, including an all-helical protein such as myoglobin, which undergoes helix-to-beta transition during thermal unfolding and aggregation^36, 37^.

**Table 1:** Secondary structure percentage of SUFU from X-ray structures and CD estimation.

	Helix	Beta strand	Other
dSUFU X-ray 4KMA	24	21	55
dSUFU CD 15 °C BestSel	20.1	21.7	58
hSUFU X-ray 4KM9	19.0	20.0	61
hSUFU CD 15° BestSel	18.5	23.6	58
zSUFU CD 15° BestSel	22.2	21.2	56

#### CD spectra of dSUFU, hSUFU and zSUFU show similar secondary structure content

The three proteins present very similar spectra at 15 °C with a maximum ellipticity at 190 nm and a minimum at 208 nm (Fig. 1C). The BestSel analysis of the spectra indicates a similar secondary structure content (Fig. 1D), confirming that they belong to the same fold family. Table 1 summarises the secondary structures contents as seen in the X-ray structures of dSUFU and hSUFU and as estimated from the CD spectra by BestSel for hSUFU, dSUFU and zSUFU. All indicate an equivalent amount of helix and beta strand, showing that the solution secondary structure is equivalent to that of the X-ray structures and that zSUFU has a similar spectrum as hSUFU and dSUFU.

### Characterisation of SUFU zinc binding properties

#### Bioinformatic analysis discloses a potential metal binding site in SUFU

**Figure 2:**
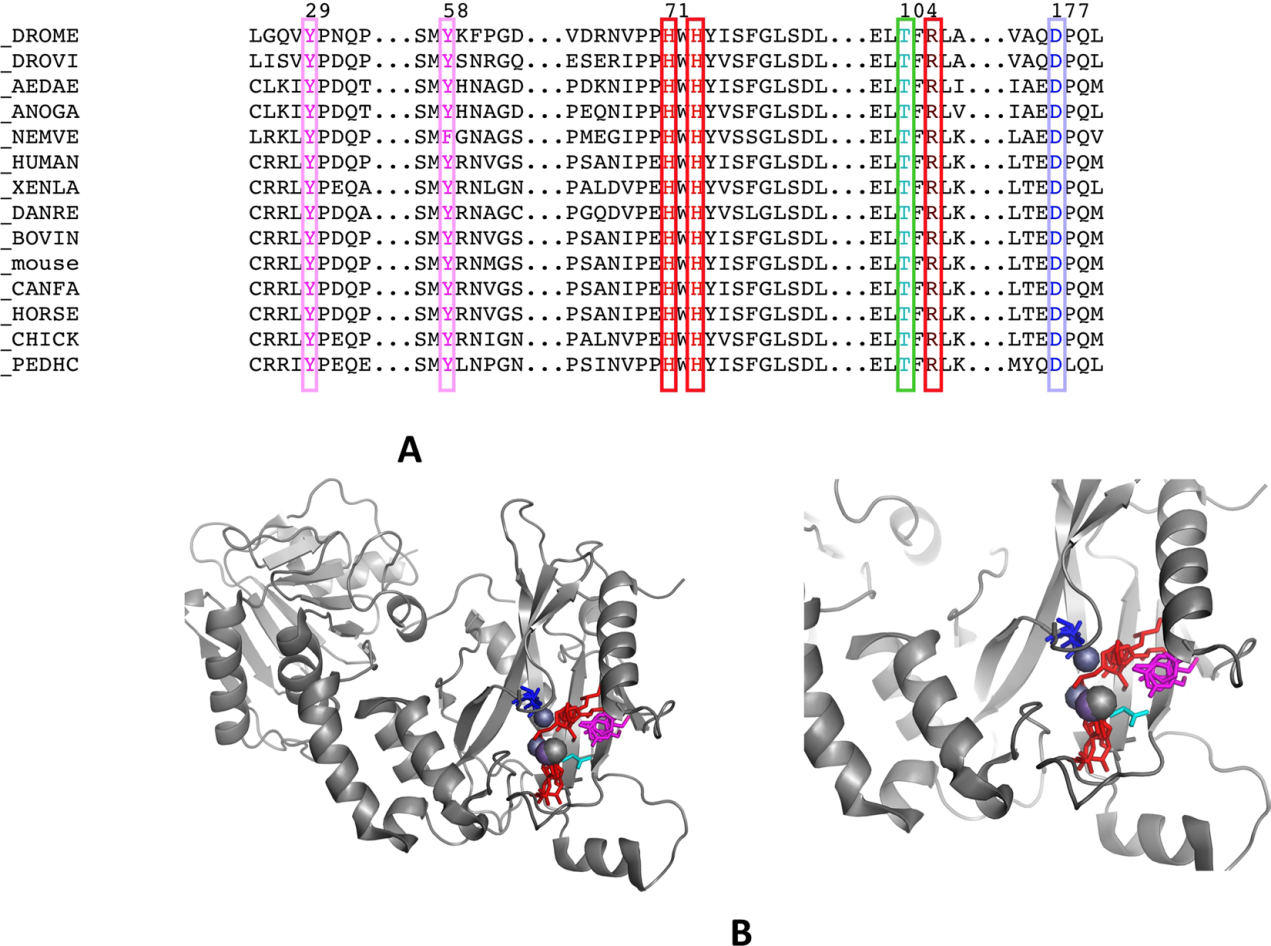
Conserved sequence and HWHY pocket in SUFU structure. (**A**) Part of aminoacid sequence alignment showing residues involved in the putative zinc-binding pocket. The numbers at the top correspond to Drosophila sequence. Conserved aminoacids are boxed and coloured as in Fig. 2B. Sequences are named using Uniprot entry code followed by species abbreviated name. DROME, *Drosophila melanogaster*; DROVI, *Drosophila virilis*; AEDAE, *Aedes aegypti*; NEMVE, *Nematostella vectensis*; HUMAN, *Homo sapiens*; XENLA, *Xenopus laevi*; DANRE, *Danio rerio*; BOVIN, *Bos taurus*; MOUSE, *Mus musculus*; CANFA, *Canis familiaris*; HORSE, *Equus caballus*; CHICK, *Gallus gallus*; PEDHC, *Pediculus humanus* subspecies corpori. (**B**) Global view and close-up of dSUFU crystal structure 4KMA showing the superposition of several cation binding sites selected by ASSAM server. The main chain is represented as a grey cartoon; the ions are displayed as spheres and conserved aminoacid side chains are coloured as follows: His, red; Asp or Glu, blue; Tyr, magenta; Thr, cyan.

A multiple alignment of SUFU amino-acid sequences from various species revealed several conserved motifs. One of them is a remarkably conserved H_71_WH_73_Y motif (residue numbering is according to the Drosophila sequence) featuring a pair of Histidine residues that point into a site that includes several other conserved residues: Y_29_, T_104_, R_107_, D_178_ and Q_180_, which are all located in the N-terminal domain and buried in the protein core (Fig. 2A for the sequence alignment and Fig. 2B for the 3D structure). We used the coordinates of those conserved residues as the input into ASSAM^38^, a program that searches the protein database for sites with similar chemical features: 89 sites belonging to 52 individual proteins, and containing a structural arrangement of 4 aminoacids with an RMSD less than 1.8 Å relative to those in SUFU, were identified. Among these sites, many involved two histidines and interacted with a ligand. The ligands are diverse, but a large majority of them are divalent cations: Mg^2+^, Fe^2+^, Zn^2+^, etc., thus indicating that SUFU may bind a metal ions at this site. This prompted us to search for a potential metal bound to SUFU.

#### Spectroscopic measurements show that SUFU co-purifies with zinc

**Figure 3:**
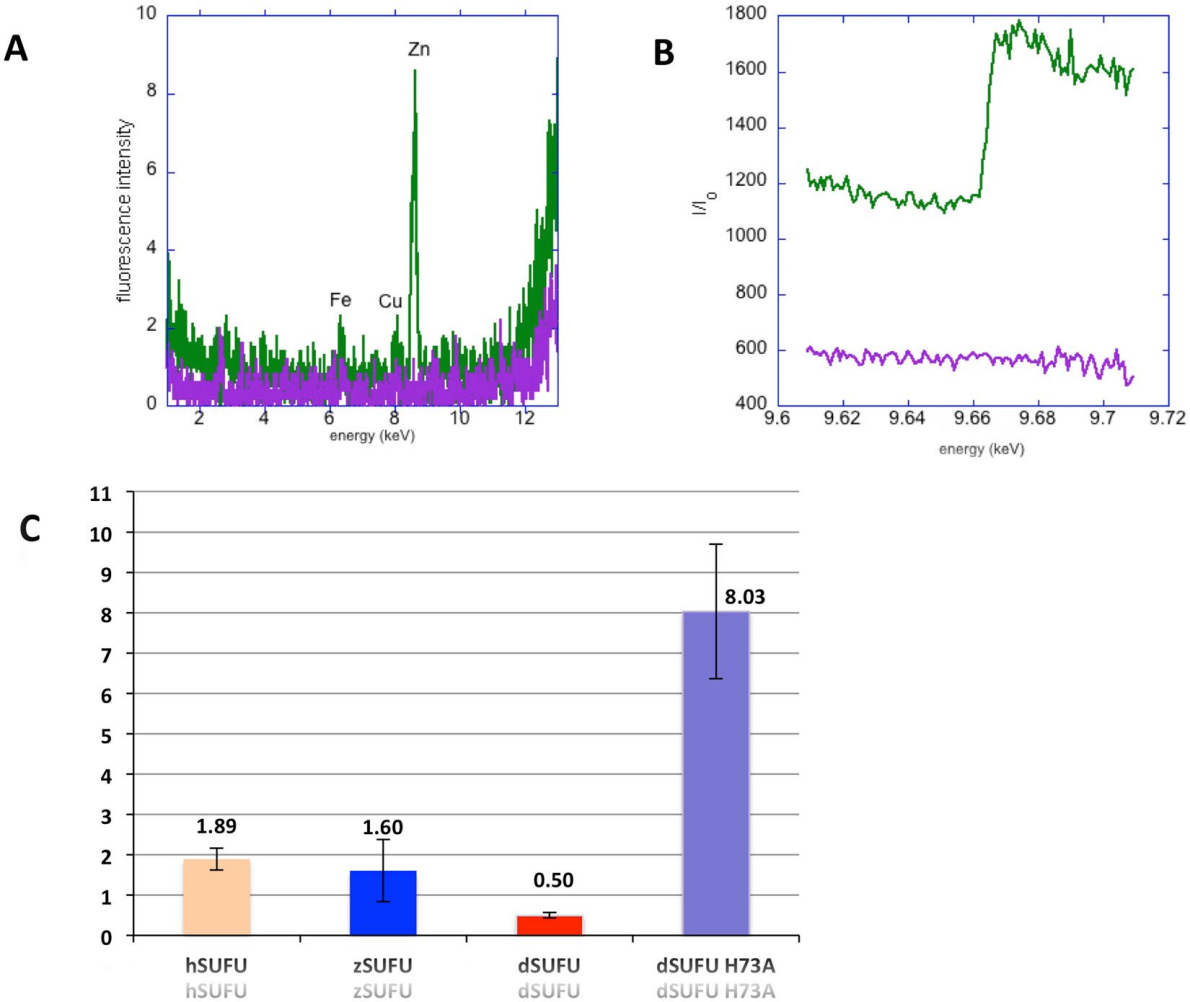
Zinc binding measurements. (**A**) X-ray fluorescence spectrum in the 0–12 keV range. (**B**) X-ray absorption spectrum shows the presence of zinc in dSUFU. In both figures, the protein spectra are shown in green and the buffer is purple. (**C**) Dissociation constant of zinc (in nM) for hSUFU (orange), zSUFU (blue), dSUFU wild type (red) and dSUFU His73A mutant (purple).

The X-ray fluorescence spectrum measured on a very concentrated (30 mg/ml) solution of dSUFU, expressed and purified with no added metal, showed the presence of an absorption edge at 9.66 keV, characteristic of the Zinc K level (Fig. 3A). Zinc was also detected using a more precise absorption spectrum around the energy of Zinc K edge, showing a clear absorption jump (Fig. 3B). In both cases, the buffer showed no trace of any metal. The presence of Zinc was also confirmed by plasma induced atomic emission spectroscopy, with a stoichiometry of 0.3 Zinc per protein monomer. Together, these measurements show that dSUFU collects zinc during expression, probably from the cell growth medium.

#### SUFU binds zinc with nanomolar affinity and histidine mutant has a lower affinity but a similar structure

**Figure 4:**
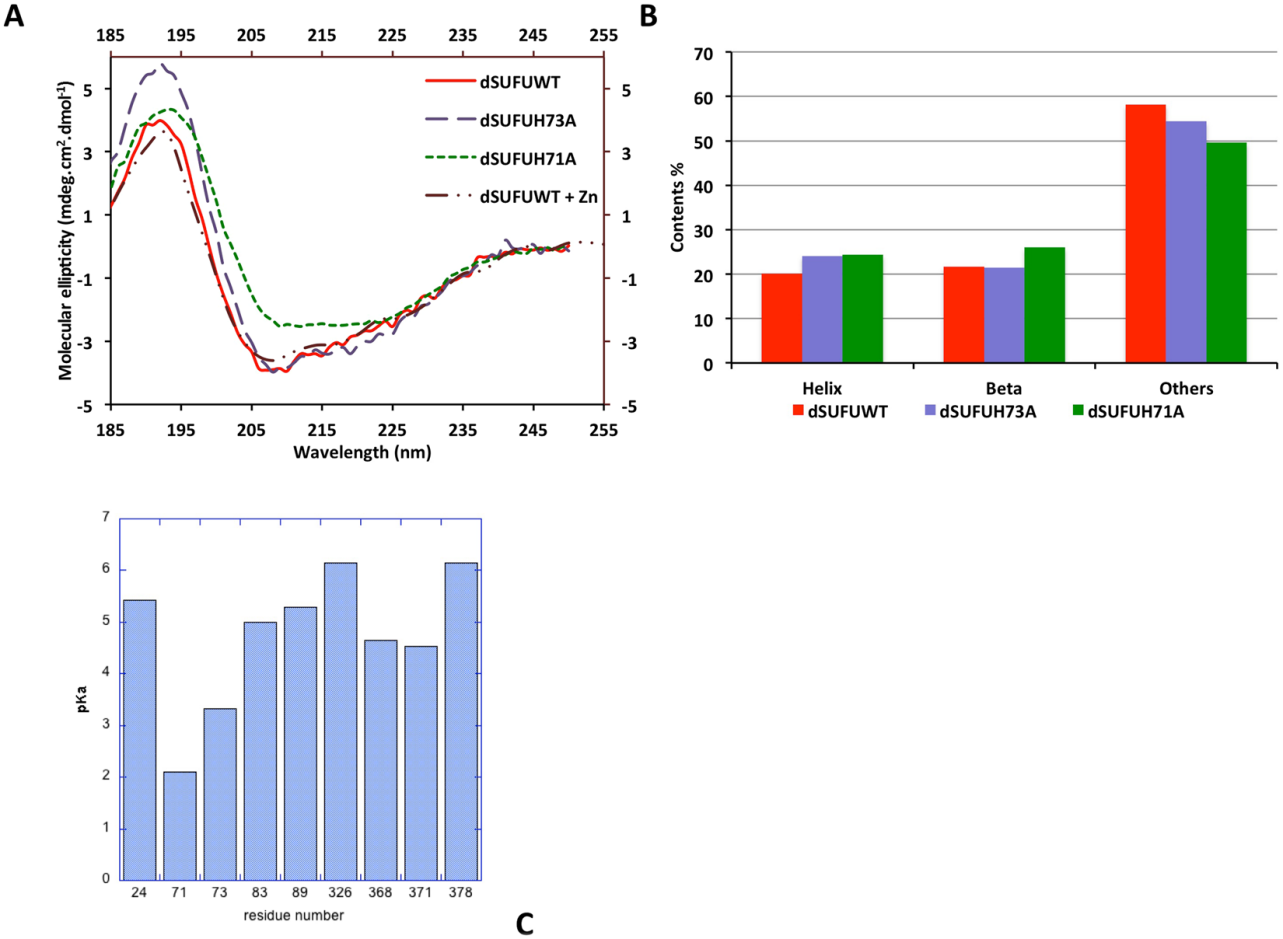
CD spectra of dSUFU with zinc added and mutants and calculated pKa of Histidine residues. (**A**) CD spectra of dSUFU wild type (red solid line), His71A mutant (green dotted line), His73A mutant (purple hashed line) and dSUFU with zinc added (brown mixed line); (**B**) Secondary structure estimation using Bestsel analysis of CD spectra of dSUFU wild type, His71A and His73A mutants (same color code as A). (**C**) Histidine residues pKa calculated using server propka.org on dSUFU structure 4 KMA.

The affinity of SUFU for zinc was measured using the 4-(2-pyridylazo) resorcinol (PAR) reagent with Drosophila, Human and Zebrafish SUFU. The affinity constant was calculated using equation 1 given in the Experimental Methods section. As shown in Fig. 3C, the zinc affinity of wild-type dSUFU, zSUFU and hSUFU are measured at Kd values of 0.5, 1.6 and 1.9 nM, respectively, showing that SUFU from the three species bind zinc with similar affinity. These values are intermediate relative to the affinity of other zinc binding proteins. For example, zinc-finger protein Metal responsive element-binding Transcription Factor MTF1 has an affinity of 30 pM^39^, carbonic anhydrase II, an important enzyme using zinc as a cofactor, has an affinity of 0.8 pM^40^, and rhodopsin, which uses zinc as a structural element, has an affinity of 0.1 µM^41^. Finally, addition of zinc does not seem to perturb the secondary structure as shown by a very similar SRCD spectrum between zinc-free and zinc-added dSUFU (Fig. 4A).

As many zinc-binding sites involve histidines and this binding is pH-dependent, we investigated the influence of pH on the binding. dSUFU has 9 histidine residues, hSUFU has 17 and zSUFU has 19. Amongst those, Histidine 71, 73, 89 and 371 (numbered according to dSUFU) are conserved in the three species. In order to further test the hypothesis of the histidine involvement in zinc binding, we lowered the pH to a value below 6.5, which corresponds to the histidine side chain theoretical pKa. As PAR loses its metal binding properties at pH values lower than 7.5, we used atomic absorption spectroscopy to monitor the amount of zinc bound to SUFU at different pH values. As shown in Table 2, SUFU co-purifies with a small, but significant, amount of zinc that does not depend on the pH. Then when incubated with 10 zinc atoms per monomer and dialysed to remove excess zinc, SUFU binds more zinc at pH 7 and 8 than at pH6. Given that the Histidine pKa values is 6.5, most histidine side chains are likely to be protonated at pH6 and thus lose their zinc binding properties. Our result is compatible with an involvement of histidines in zinc binding.

**Table 2:** Number of zinc atoms bound per protein molecule.

	dSUFU	dSUFU + zinc
pH 6	0.23 ± 0.05	4.4 ± 0.38
pH 7	0.20 ± 0.04	6.7 ± 0.65
pH 8	0.39 ± 0.02	6.9 ± 0.46

To probe the importance of the two histidines in the H_71_WH_73_Y site of the Drosophila sequence, we replaced them by alanine and expressed them in the same bacterial conditions as the wild type protein. All proteins were expressed in the same amount and similar soluble proportion as the wild type as shown with an anti-his tag western blot (Supplementary Figure 1A). The double mutant was however extremely unstable and we could not purify enough soluble protein to analyse it, which thus demonstrates that this region is important for SUFU stability. Mutant H71A was also quite unstable, albeit less so than the double mutant, and only enough material could be purified for CD measurements. Conversely, mutant H73A behaved like the wild-type protein in terms of both expression and purification yields. CD spectra (Fig. 4A) show that the H73A mutant had a similar fold as the wild-type protein, whereas H71A had a much shallower spectrum than the wild type protein and H73A mutant at 208 nm, indicative of a lower content of regular secondary structure. Surprisingly, Bestsel analysis (Fig. 4B) denotes a similar content in helix and beta structures to that of the wild-type protein (Fig. 1B). We could not calculate the zinc dissociation constant for H71A because a reliable titration could not be performed due to lack of protein. In contrast, it was possible to calculate a dissociation constant for H73A, which at 8 nM is quite high, but still 16 times lower than for the wild-type protein. This decrease in affinity is much less drastic than that observed for carbonic anhydrase which loses a factor of 10^4^ in affinity for zinc upon mutation of Histidine 94 to Alanine^40^. We then expressed the wild type and mutant SUFU in drosophila CL8 cells (that are known to respond to HH), using the same amounts of vector. We found that they are present in the same amount, showing that the mutations do not change their stability in drosophila cells similarly to bacteria (Supplementary Figure 1B). It seems that the wild type and mutants only show stability differences during purification.

In summary, SUFU from three species is capable of binding zinc in a pH - dependent manner. In the presence of an excess of zinc, it binds four ions at pH 6 and six ions at pH 7 and 8 per monomer.

#### Analysis of a potential Zinc binding site

The crystal structure of SUFU was then analysed to determine whether it could accommodate a zinc ion at the site of the conserved pocket including His71 and His73 found by the ASSAM program. The presence of two Histidines separated by only one aminoacid is rather rare in protein sequences (30049 HXH hits vs. 202957 AXA hits in human sequences where X is any aminoacid, H is histidine and A is alanine). Amongst those, it is difficult to know how many bind zinc. However, a survey of the BioLiP database (http://zhanglab.ccmb.med.umich.edu/BioLiP) for proteins binding one Zn^2+^, revealed 311 unique PDB entries with an HXH sequence of the total 9422 zinc binding structures in the PDB. The environment around His71 and His73 in SUFU (i.e., the presence of Tyr60, Gln180 and Asp178), constitutes a pocket that can accommodate an ion such as Zn ++ . Moreover, pKa calculation using pdb2pka.org server on dSUFU structure 4KMA showed that the pKa of His71 and His73 are 2 and 3.2, respectively (Fig. 4C). pKa calculation of hSUFU histidines gives similar results (not shown). These are considerably lower pKa values than the average pKa value of 6.5 for Histidine.

A review article by Alberts *et al*. disclosed that Zinc binding in protein X-ray structures occured most often via histidines but other binding sites included acidic residues Glu and Asp^42^. Examination of SUFU structures revealed several other conserved pockets. In particular, His83, His89 and Asp53 are all bound to the same water molecule. This site is complemented by Val88. All residues are conserved and could constitute a zinc-binding site. In addition, several acidic residues are conserved between hSUFU and dSUFU, but it would be very speculative to predict which ones could form a zinc-binding site. In conclusion, the site harboring conserved His71 and His73 is appropriate for zinc binding, but it is probably not the only one.

## Discussion

### SUFU binds zinc

Two grams of Zinc are present in an average human body, making it the second most abundant transition metal in the organism, and its physiological importance is underscored in its structural influence in proteins such as zinc fingers^43^, as well as its regulatory role in many metabolic enzymes such as carbonic anhydrase^30, 44^. We discovered that SUFU binds zinc in a manner not modifying its secondary structure and that one of the binding sites may involve two conserved histidines from the N-terminal domain. This was a surprise because, i) SUFU does not present a canonical zinc-binding site and, ii) no zinc is visible in any SUFU crystal structure. Even after careful re-examination of electron density maps calculated using PDB deposited coordinates and structure factors, we were unable to detect any zinc atom(s) in the crystal structures of hSUFU and dSUFU, even though the pocket containing the HWHY motif is particularly well ordered, with temperature factors 20% lower than the mean of the structure^27, 28^. In most structures, either EDTA or tartrate are present in the protein solution, or in the crystallisation conditions, and may therefore have chelated cations during the several day - long crystallisation process. Two crystal structures of a construct composed of a 6-Histidine tag, hSUFU and Maltose-Binding Protein were obtained by Cherry *et al*., in the presence of one zinc equivalent per protein monomer (PDB entries 4BLB and 4BLD)^27^. The zinc is bound in a non-physiological site at the interface between the 6-Histidine tag of one protein and the Maltose-Binding Protein tag of another chain. Therefore, the nine deposited structures of SUFU are devoid of zinc, most probably due to crystallisation conditions. Alternatively, it is also possible that the occupancy of zinc taken from the growth medium is too low to be seen by diffraction. Indeed, the background zinc binding of 0.3 atom per protein monomer that we observed with atomic emission spectroscopy and colorimetric measurements may be distributed over several binding sites. A valid comparison can be made with carbonic anhydrase, an enzyme that binds zinc with high affinity in a site containing three histidines but remains structured and stable when zinc is removed by dialysis against a chelating agent over a period of days^45^.

We identified a conserved aminoacid motif around His71-His73 and showed that mutating those histidines results in similar expression in *E. coli* and drosophila cells but that zinc affinity is reduced when His73 is mutated into alanine, and that the amount of zinc bound to SUFU is lower at pH below the pKa of histidine, indicating that this residue might be involved in one of the metal binding sites. A possible explanation may be that, in the absence of His73, part of SUFU uses His71 and Gln180 as an alternative ligand for zinc, thereby retaining part of this mutant’s affinity for zinc. pKa calculation for histidine residues showed that His71 and H73 have much lower pKa than other residues. This may explain i) the destabilisation of purified SUFU upon mutation of His71 and double mutation, and ii) the relative decrease of affinity for zinc of the H73A mutant. As shown in Fig. 4C, the other histidine residues have higher pKa values, which may explain the increase in zinc binding observed at pH values above 6. The importance of this region is also emphasized by the observation that the *dre* mutant in zebrafish incurs overactivation of the Hh pathway due to a loss of SUFU function. This is a point mutation replacing Thr111 by a lysine^23^. This Threonine (numbered 105 in drosophila) interacts with His73 in the crystal structure of dSUFU. Its substitution by a more bulky and negatively charged lysine undoubtedly changes the pocket and probably destabilises the protein. Another mutation, replacement of Arginine 123 by cysteine was found in connection to meningioma^46^. This arginine is also present in the pocket hosting His71 and His73. Indeed, Aavikko *et al*. have expressed mutated sufu in several cell types and showed that mutated SUFU is less expressed and fails to relocate Gli in the cytoplasm. We have compared the levels of wild-type Sufu protein with mutation disrupting zinc binding (H71A and H73A mutants) in extracts of Cl8 cells that were transfected with exactly the same levels of vectors. However, we could not detect any difference. This was reproduced twice. It is in agreement with the similar expression level and solubility of the mutant forms purified from *E. coli*. Therefore, the lower zinc binding affinity of H73A mutant that we observe is not due to a misfolding but shows the importance of this histidine in the binding.

Zinc homeostasis is crucial for the HH pathway. Zinc is necessary for the formation of complex between Hedgehog-interacting protein and Sonic hedgehog^47, 48^, and zinc depletion leads to an activation of the HH pathway via SHH intein cleavage^49^. Zinc balance may also be important for intracellular partners of the HH cascade. Inside the cell and in the nucleus, SUFU interacts with CI/Gli, a five zinc-finger transcription factor. In another case, the zinc-binding DNA binding domain of p53 needs to be supplied with zinc, but too high a concentration is deleterious for its proper folding, resulting in aggregation^50^ and zinc concentration can be buffered by zinc binding proteins such as metallothionein^51^. Thus, in a tightly regulated pathway such as HH, zinc bound to SUFU could modulate CI/Gli structure or select CI/Gli gene target in the nucleus^52^.

Our work presents a novel finding, the zinc binding property of SUFU, which so far has remained undetected. Further studies are needed to characterise the role of zinc on the affinity between SUFU and its partners and also the possible phenotypic changes of mutants affecting zinc binding in the cell.

## Methods

Protein structures are displayed with Pymol v. 1.2 (DeLano scientific, 2009). Unless otherwise stated, residue numbering is according to dSUFU Uniprot entry Q9VG38.

### SUFU Cloning, Expression in bacteria, and Purification

The dSUFU gene was cloned using the Gateway technology (Invitrogen), into the pDEST17 (Invitrogen) expression vector according to standard Gateway^TM^ protocols. The final construct encoded an N-terminal hexahistidine tag and a TEV protease cleavage site before the gene of interest. Mutants His71A and His73A of dSUFU were obtained by site-directed mutagenesis using the quikchange protocol (Invitrogen). The resulting plasmid was used to transform *E. coli* strain C41 (DE3)^53^. Cells were grown in 2YT medium at 37 °C until the optical density reached 0.6. Protein expression was induced with 0.2 mM isopropyl β-thiogalactoside overnight at 20 °C. Bacterial cells were recovered by centrifugation at 5000 g for 15 min and frozen at −80 °C. Similar procedures were used for hSUFU and zSUFU, which were cloned in a pACYDuet vector.

Lysis was achieved in buffer A (50 mM Tris pH 8.0, 500 mM NaCl, 5 mM imidazole, 5% glycerol). Cells were thawed with shaking in the presence of 5 mM MgCl2 and 10 μg/ml of DNase for 1 hour then lysed with Constant cell disruptor at 2 kbar. Soluble proteins were separated from inclusion bodies and cell debris by a 1-hour centrifugation step at 9000 g. The supernatant was loaded on a 1 mL HisTrap nickel affinity column (Roche) equilibrated in buffer A. The column was washed with buffer A and eluted with a 5–300 mM imidazole gradient. The eluted protein was dialysed against buffer B (10 mM Tris pH 8.5, 100 mM NaCl, 5% glycerol, 2.5 mM MgCl_2_ and 2 mM β-mercaptoethanol) for 12 + 2 hours, then incubated with Tobacco Etch Virus protease for 24 hours at room temperature to cleave the 6-Histidine tag and the associated linker. The cleavage mixture was then dialysed back into buffer A and incubated with Ni sepharose (GE Healthcare) resin for 1 hour so that uncleaved protein, the TEV protease and the 6-Histidine-tag can be separated from the cleaved protein. The flow through was then concentrated and injected on a preparative HiLoad 16/91 XK Superdex 200 (GE Healthcare) size exclusion column in a buffer containing 10 mM Tris pH 8.0, 100 mM NaCl, 5% glycerol, 2 mM β-Mercapto-ethanol. Purified material was appropriately concentrated on a Vivaspin (Sartorius) centrifugal filter unit with a 10-kDa cutoff; aliquoted, and frozen in liquid nitrogen for further use.

### Circular dichroism measurements

Synchrotron radiation circular dichroism (SRCD) spectra were measured at the DISCO beamline^54^ (SOLEIL synchrotron source at Saint Aubin, France). Aliquots of SUFU were dialysed against phosphate 20 mM pH 7.5 and NaF 50 mM. All sample concentrations were measured using the absorbance at 280 nm, after centrifugation and just prior to measurement, using a theoretical extinction coefficient calculated using Protparam server http://web.expasy.org/protparam/. All concentrations were between 4 and 6 mg/ml.

The beamline monochromator was calibrated using a camphorsulfonic acid solution prior to measurements. Four microlitres of sample was deposited on one face of a calcium fluoride circular cuvette (Hellma), then the second face was carefully positioned and the cuvette closed by capillary force. Interferometry measurement showed the optical path to be 12 µm. The cuvette was placed in an airtight, metal sample holder that was positioned in the beamline Peltier temperature controlled chamber, allowing for quick temperature changes with very little evaporation. Spectra were measured between 280 and 170 nm, each final spectrum averaged from three measurements. Thermal unfolding measurements were performed by averaging three spectra collected in steps of 5 °C between 15 and 95 °C. Buffer spectra were taken in the same conditions for subtraction from the protein spectra. Spectra were then processed for buffer subtraction and scaling using the CDTools program^55^. Secondary structure decomposition was done using the program BestSel^35^.

### Search for 3D sites with similar aminoacid organisation

A multiple alignment of SUFU aminoacid sequences disclosed a number of conserved motifs, amongst which, one with sequence H_71_WH_73_Y that is universally conserved in dSUFU sequences. Examination of the protein structure showed that this site is buried in the protein and in contact with other conserved residues Y29, T105, R107 and D178 forming a potentially interesting pocket (see Fig. 2B). The PDB coordinates of the corresponding residues Y45, H87, H89, T121, R123 and D182 from structure 1M1L of hSUFU was input in the ASSAM server, a program that searches the protein data bank to locate structures with similar amino-acid composition and geometry^38^.

### Metal binding site characterisation

- X-ray fluorescence and absorption measurements were performed at the Proxima1 beamline (SOLEIL synchrotron). About 1 µl of the concentrated protein (37 mg/ml or 71 µM), or its purification buffer, were scooped into a nylon loop and flash frozen at 100 K in a cryo-stream. The excitation energy used for the fluorescence scan was 15 keV, thus exciting the K or L absorption edges of most transition metals. The buffer data was subtracted from the protein spectrum and plotted. Absorption spectra were measured in a 200 eV region around the edge of interest, with 10 seconds exposure per eV.
- Atomic emission spectroscopy inductively coupled plasma - optical emission spectrometry was performed on a Varian Vistapro apparatus. Standards containing solutions of known concentration for Al, Ba, Ca, Fe, K, Li, Mg, Mn, Na, Si, Sr, Ti and Zn were first used for calibration. Then the buffer and the protein (300 µl at 0.3 mg/ml) solutions were injected and the emission spectra were analysed for quantisation of the above-mentioned ions.
- Atomic absorption spectroscopy (AAS) was performed on a Varian AA20 apparatus calibrated with a freshly diluted zinc standard solution at the appropriate pH, at concentrations between 0.625 µM and 10 µM. Several 100 µl aliquots of the protein sample at concentrations between 1 and 5 µM were injected and the absorbance peak measured.
- Affinity measurements were performed using 4-(2-pyridylazo) resorcinol (PAR) reagent on a Hewlett Packard spectrophotometer at 27 °C. Free PAR has an absorption maximum at 410 nm whereas Zinc-bound PAR has a maximum at 500 nm^56^. We started from fully ligated PAR at 10 µM with 4 µM zinc and progressively added SUFU or Ethylene glycol-bis(2-aminoethylether)-*N,N,N′,N′*-tetraacetic acid (EGTA) to titrate the zinc. The absorbance at 500 nm decreased in a hyperbolic fashion and the Kex experimental affinity constant was calculated by a least-squares fit with the Kaleidagraph programme to the equation [Zn-SUFU] = [Zn-SUFU]_max_ × [SUFU]/(Kex + [SUFU]), where [Zn-SUFU] is the concentration of the SUFU-Zn complex derived from measurement of the absorbance at 500 nm, and [Zn-SUFU]max is the maximum complex concentration measured. Kd between SUFU and Zinc was calculated using equation 1 and data listed in Table S4 from^57^;

1 $${\rm{Kd}}({\rm{Zn}}\mbox{-}{\rm{sufu}})={\rm{Kd}}({\rm{Zn}}\mbox{-}{\rm{EGTA}})\times ({{\rm{Kex}}}_{\text{Zn}\mbox{-}\text{EGTA}}/{{\rm{Kex}}}_{\text{Zn}\mbox{-}\text{Sufu}})$$

with Kex_Zn-EGTA_ = 5 µM (our unpublished results) and Kex _Zn-Sufu_ the measured experimental affinity. log Ka_pH7.5_ (Zn-EGTA) = 9.39 at pH 7.5, therefore Ka_pH7.5_ (Zn-EGTA) = 2.45 × 10^9^ and Kd = 1/Ka = 4.07 × 10^−10^ M. Therefore Kd(Zn-sufu) = 4.07 × 10^−10^ × 11/Kex Zn-sufu.

## Electronic supplementary material

Supplementary Information 
