# Supplementary material for: Biophysical characterisation of the novel zinc binding property in Suppressor of Fused
Source: Sci Rep. 2017 Sep 11;7:11139. doi: 10.1038/s41598-017-11203-2 (PMC5593987; doi:10.1038/s41598-017-11203-2)
Supplement: Supplementary file 1 — Supplementary Information [file 41598_2017_11203_MOESM1_ESM.pdf]

Title: Biophysical characterisation of the novel zinc binding property in Suppressor of Fused.

Author list
Amira Jabrani [1*]; Staëlle Makamte[1*]; Emilie Moreau[1]; Yasmine Gharbi[1]; Anne Plessis[2]; Lucia Bruzzone[2]; Matthieu Sanial[2] and Valérie Biou[1]

* both authors contributed equally to this work.
Corresponding author Valerie Biou valerie.biou@ibpc.fr

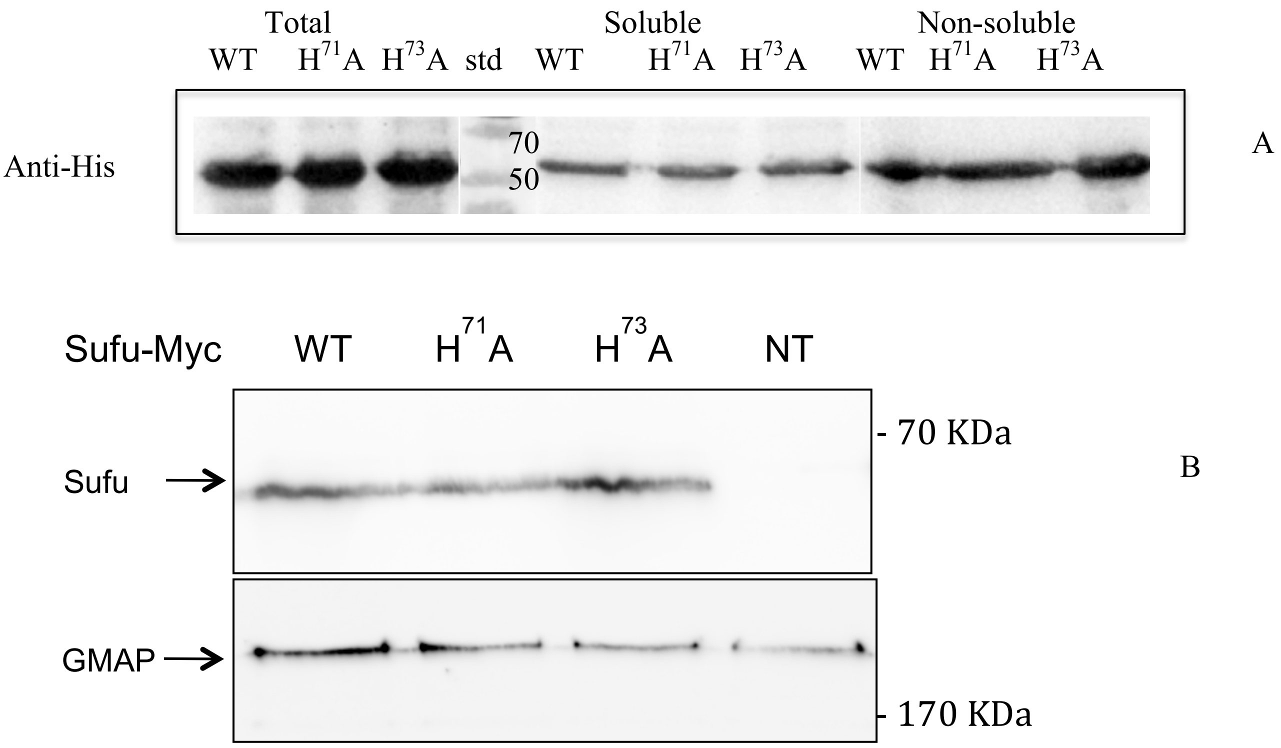

SUPPLEMENTARY FIGURE 1

Legend to Supplementary Figure 1
drosophila wild-type and mutant SUFU expression. A, bacterial C41(DE3) expression. anti-His tag western blot. WT, wild-type; H71A and H73A, mutants; 50, 70= molecular weight markers (in kDa). B, drosophila C18 cell expression. WT, wild-type; H71A and H73A, mutants, NT, not transfected.

Methods for Supplementary Figure 1
a) For the expression control in bacteria, C41(DE3) E. coli cells were transformed with pDest17 plasmids encoding dSUFU wild type and mutants with an hisdine tag at the N-terminus. An overnight preculture was grown from a single clone as mentioned above. The cell pellets were thawed, added with 30 ml buffer A and lysed. The absorbency of the suspensions was measured at 600nm and they were diluted to 1 OD. An aliquot of the total extract was kept apart and the rest was centrifuged at 9000g for one hour. The supernatant was removed and the pellet suspended in the same volume of buffer A. 15% acrylamide SDS-PAGE gels were run with 25 µl of each sample, transferred onto nitrocellulose membrane at 100V for 1.5 hours and exposed overnight to 1:1000 anti-his antibody (His-tag mAB New England Biolabs) then for one hour to 1 :10000 dilution of anti-mouse HRP conjugate secondary antibody (Promega) and revealed by chemiluminescence on a Biorad Chemidoc.
b) Drosophila cell expression
Cl8 cells culture, transfection, protein extraction and dosage were performed as described in [1]. The equivalent of 60 µg of protein was heated for 3 minutes at 95°C before loading on a 12% Anderson gel. The gels were run using a miniprotean apparatus (Bio-Rad) for 90 minutes at 150 volts (constant voltage). The proteins were then transferred for 75 minutes at 100V onto Nitrocellulose membrane (0,2µM, Protran BA 83 Schleicher & Schuell) using the same apparatus and blotted with 1:2000 Rabbit anti-GMAP (Sigma), 1:1000 Mouse anti-Myc (clone 4A6, Millipore), secondary antibodies conjugated with HRP: anti-Rabbit (JacksonImmuno) and anti-Mouse (Sigma). The immunolabeled bands were detected with the enhanced chemiluminescence detection system (ECL Select, Amersham) on a LAS-3000 imager (Fujifilm).

Reference

1. Sanial, M. *et al.* Dose dependent transduction of Hedgehog relies on phosphorylation-based feedback between the GPCR Smoothened and the kinase Fused. *Development* **144,** 1841–1850 (2017).
